# Longitudinal Peripheral Blood Transcriptomics Reveal Novel Signatures During Cardiac Allograft Rejection

**DOI:** 10.1097/TXD.0000000000001882

**Published:** 2026-01-20

**Authors:** Rachad Ghazal, Min Wang, Akshatha N. Srinivas, Jenny J. Cao, Hridyanshu Vyas, Duan Liu, Asha Nair, Byron H. Smith, Li Wang, Daniel S. Yip, Parag C. Patel, David E. Steidley, Brian W. Hardaway, Alfredo L. Clavell, Sudhir S. Kushwaha, Walter D. Park, Mark D. Stegall, Howard J. Eisen, Naveen L. Pereira

**Affiliations:** 1 Department of Cardiovascular Medicine, Mayo Clinic, Rochester, MN.; 2 Department of Molecular Pharmacology and Experimental Therapeutics, Mayo Clinic, Rochester, MN.; 3 Department of Computational Biology, Mayo Clinic, Rochester, MN.; 4 Department of Cardiovascular Medicine, Mayo Clinic, Jacksonville, FL.; 5 Department of Cardiovascular Medicine, Mayo Clinic, Phoenix, AZ.; 6 Department of Surgery, Mayo Clinic, Rochester, MN.; 7 Divison of Cardiology, Thomas Jefferson University Hospital, Philadelphia, PA.

## Abstract

**Background.:**

Acute cellular rejection (ACR) remains a major cause of morbidity after heart transplantation despite advances in immunosuppression. Whole genome transcriptomic profiling offers a systems-based, unbiased approach to elucidate the molecular mechanisms underlying ACR. However, noninvasive, longitudinal biomarker assessments capable of capturing the temporal dynamics of rejection biology remain scarce.

**Methods.:**

RNA sequencing of peripheral blood from heart transplant recipients before, during, and after ACR was compared with nonrejection controls. Pathway analysis was conducted using differentially expressed genes (DEGs), and a machine learning approach was applied to assess gene-based prediction of ACR.

**Results.:**

A total of 235 rejection-specific significant DEGs and 863 postrejection DEGs (false discovery rate < 0.05) were identified. During ACR, DEGs were enriched for T-cell activation/differentiation, apoptosis, and B-cell receptor signaling pathways. By combining the 2 sets of DEGs, a panel of 71 common genes was identified that reflected the significant, longitudinal transcriptomic dynamics of ACR. In an elastic net machine learning–based classifier, *DYNLL1* and *SERF2* were identified as ACR predictive genes, and achieved a cross-validated area under the receiver operating characteristic curve of 0.63.

**Conclusions.:**

Peripheral blood transcriptomics identify dynamic temporal responses in ACR including T- and B-cell pathways with potential ACR predictive genes that warrant further investigation.

## INTRODUCTION

Acute cellular rejection (ACR) continues to be a significant complication after heart transplantation (HTx).^1^ ACR is an important cause of hospitalization and graft dysfunction especially during the early years post-HTx. Therefore, developing better approaches to understand the changes that occur during ACR could lead to a better understanding of the pathogenesis of ACR with diagnostic and therapeutic implications. Peripheral blood mononuclear cells transcriptomic profiling that analyzes the expression of 20 targeted genes has been used to detect cardiac rejection^2^ but such a targeted approach in the absence of a longitudinal analysis to detect differentially expressed genes (DEGs) does not allow the identification of important biological pathways involved in rejection. The advent of whole genome transcriptomic profiling of peripheral blood allows such a longitudinal comparison of global gene expression patterns in HTx recipients before, during, and after ACR, providing an unbiased approach to identify genes and pathways involved in rejection. Longitudinal studies offer valuable mechanistic insights and, by comparing individuals to themselves over time, help detect biomolecular responses by accounting for personal variation—one of the major sources of variability in data analysis.^3^ In the present study, we used peripheral blood whole genome transcriptomic profiling to describe not only the dynamic changes that occur in gene expression during and post ACR and but also identify by a machine learning approach, unique genes that may predict the development of ACR.

## MATERIALS AND METHODS

### Study Population

A multicenter case-control study design was chosen, in which 100 patients who underwent HTx were enrolled between November 2017 and July 2019. Peripheral blood samples of these adult HTx recipients were collected at Mayo Clinic (Minnesota, Arizona, and Florida) at 1, 3, 6, and 12 mo post-HTx. Routine endomyocardial biopsy (EMB) was performed weekly for the first 6 wk after transplant beginning 1 wk after completion of induction therapy, then every 2 wk between 6 wk and 3 mo, monthly between 3 and 6 mo, and then every 3 mo until the end of the second year, in addition to being performed for clinical symptoms or signs suggestive of rejection. EMB was performed using standard technique. The diagnosis of ACR was made based on International Society for Heart and Lung Transplantation (ISHLT) criteria. Biopsy interpretation was performed by local pathologists at each participating center. We included both ISHLT grade 1R and 2R rejection to capture the spectrum of alloimmune responses. Grade 1R, though not routinely treated, represents measurable alloimmune activity that provides insight into rejection. Clinical parameters including left ventricular ejection fraction at time of biopsy, cardiac biomarkers, and documented infections within 30 d of sampling were collected from medical records. This study was approved by the Mayo Clinic Institutional Review Board. All patients provided written informed consent for blood collection, result analysis, and access to their medical records for research purposes.

### RNA Sequencing

Samples were prepared for RNA sequencing using Illumina’s TruSeq RNA Exome preparation method from PAXgene. The quality of extracted RNA was assessed using an RNA Integrity Number and a DV200 value generated by the Agilent Fragment Analyzer. Quantitative concentration was determined using a Qubit fluorometer. Samples isolated from Qiagen PAXgene Blood RNA System were treated with Zymo Research DNase I followed by an RNAClean XP (Beckman Coulter). Treated RNA was then assessed for purity and concentration using Thermo Fisher Nanodrop.

RNA Exome libraries were prepared per manufacturer’s instructions to generate cDNA libraries with an input of 50 ng. These cDNA libraries were then pooled ranging from 1-plex to 4-plex and normalized to 200 ng/sample and the transcriptome coding regions were captured. Final pools were assessed for quality using both Agilent Bioanalyzer and Qubit. Pooled libraries were then sequenced on Illumina NovaSeq 6000 at 75 million read pairs per sample following Illumina’s standard protocol on an S2 flow cell. S2 flow cells were sequenced at 2 × 100 bp paired-end reads using a NovaSeq S2 sequencing kit, NovaSeq Control Software v1.7.5 and base-calling was analyzed using Illumina’s RTA (v3.4.4).

### Bioinformatics Analysis

The raw RNA sequencing paired-end reads for the samples were processed through the Mayo RNA-Seq bioinformatics pipeline, MAP-RSeq (v3.1.4).^4^ Briefly, MAP-RSeq employs the very fast, accurate and splice-aware aligner, STAR,^5^ to align reads to the reference human genome build hg38. The aligned reads were then processed through a variety of modules, such as gene expression quantification, gene fusion detection, single nucleotide and indel detection, etc. in a parallel fashion. Gene and exon expression quantification were performed using the Subread package^6^ to obtain both raw and normalized (Fragments Per Kilobase of transcript per Million mapped reads) reads. Comprehensive analyses were also run on the aligned reads to assess quality of the sequenced libraries. Results from all modules were collated into a single HTML report by MAP-RSeq. Due to the nature of the sequencing library protocol (TruSeq RNA Exome), genes reported from MAP-RSeq were further subset to those annotated as protein-coding according to Ensembl gene definition (v78). The 21 983 protein-coding genes were used for further analysis of differential gene expression between groups. Mean expression of candidate genes in each group were considered when generating heat maps showing differential expression data.

### Differential Expression and Pathway Analysis

Using the raw expression counts of protein-coding genes from MAP-RSeq, genes differentially expressed between the groups were assessed using the bioinformatics package edgeR (v2.6.2).^7^ Genes found different between the groups were reported along with their magnitude of change (log2 scale) and their level of significance (false discovery rate [FDR] < 0.05). Using DEGs, gene set enrichment analysis was performed using the BioPlanet^8^ pathway collection from the Enrichr database^9^ to identify pathways of significance. Multiple test correction using the Benjamini-Hochberg procedure was applied to select statistically significant pathways at adjusted *P* value <0.05 from BioPlanet.

### Classifier Construction

Logistic regression with an elastic net penalty (80% L1 penalty and 20% L2) was used to perform variable selection to model rejection status based on expression of 233 genes using the “glmnet” package. Final models used unpenalized (“relaxed”) coefficients for the variables that remained in the model. Models were evaluated using the overall area under the receiver operating characteristic curve (AUC) as well as a bootstrapped estimate of AUC. 5-fold cross-validation was used to optimize the penalty used in the regression process, both for the final model as well as during the bootstrapping process. All analyses were performed in R v4.1.2 (R Foundation for Statistical Computing, Vienna, Austria).

### Protein–Protein Interaction Network of the DEGs

Protein–protein interaction (PPI) networks were generated in Cytoscape^10^ using the stringApp plugin (confidence ≥0.9; ≤20 additional interactors). EnrichmentMap was then used to visualize functional modules and their relationships.

### Statistical Analysis

Clinical characteristics were expressed as mean ± SD for continuous variables and percentages for discrete variables. Continuous variables were compared with Student *t*-test or Mann-Whitney U test, as appropriate. Categorical variables were compared with chi-square or Fisher exact tests. Normality was assessed with the Kolmogorov–Smirnov test.

## RESULTS

### Study Population Baseline Characteristics

**FIGURE 1.**
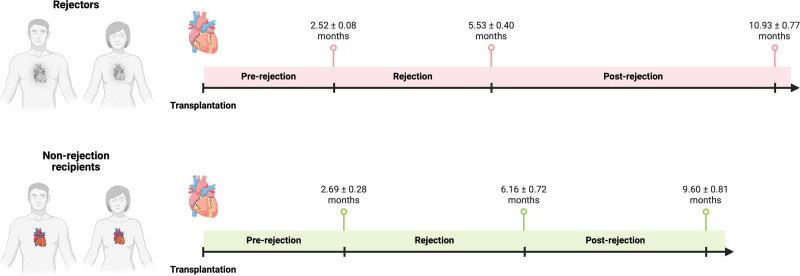
Sampling timepoints. Whole blood samples of rejectors or NR recipients from the timepoints of rejection, pre-rejection and postrejection were applied to RNA extraction followed by RNA-seq. Blood collection at rejection timepoint occurred at 5.53 ± 0.40 mo after transplantation in rejectors and 6.16 ± 0.72 mo NR controls. Timepoint of pre-rejection in rejectors vs NR recipients is 2.52 ± 0.08 vs 2.69 ± 0.28 mo; timepoint of postrejection in rejectors vs NR recipients is 10.93 ± 0.77 vs 9.60 ± 0.81 mo. The comparison between rejection to pre-rejection is defined as comparison 1 and the comparison between postrejection to rejection is defined as comparison 2. NR, nonrejection.

Among the initial 100 patients enrolled, 18 patients diagnosed with ACR (67% males) were selected as cases after matching for age, sex, and time post-HTx, and 12 matched patients without ACR served as controls. There were 6 recipients with ISHLT ≥grade 2R and 12 recipients with ISHLT grade 1R rejection. Table 1 summarizes the baseline characteristics of the study population. The mean age at transplantation was 57.67 ± 10.96 y (rejectors, 57.33 ± 13.33 y; controls, 58.17 ± 6.46 y; *P* = 0.843), and 66.7% (rejectors, n = 12, 66.7%; controls, n = 12, 66.7%; *P*p = 1.000) were male. The donor mean age was 29.70 ± 11.20 y (rejectors, 29.00 ± 10.11 y; controls, 30.75 ± 13.74 y; *P* = 0.683). Mean time of blood collection after transplantation was 5.91 ± 0.47 mo at the time of rejection (rejectors, 5.53 ± 0.40 mo; controls, 6.16 ± 0.72 mo; *P* = 0.843); prerejection, 2.62 ± 0.19 mo (rejectors, 2.52 ± 0.04 mo; controls, 2.69 ± 0.28 mo; *P* = 0.965); postrejection, 10.17 ± 0.59 mo (rejectors, 10.93 ± 0.77 mo; controls, 9.60 ± 0.81 mo; *P* = 0.789) (Figure 1). Clinical parameters showed no significant differences between groups. Mean left ventricular ejection fraction was preserved in both rejectors (58 ± 7%) and controls (60 ± 5%, *P* = 0.42). No patients had documented bacterial or viral infections within 30 d of blood sampling. Troponin levels were within normal limits for all subjects. Induction therapy and baseline immunosuppression were not significantly different at 3- and 12-mo posttransplant (Table 1). Patients with clinically significant rejection were treated with augmented steroid therapy.

**TABLE 1.** Baseline characteristics of patients

	Rejection	Control	Total	*P*
n	18	12	30	
Age at transplantation, y (range)	57.33 ± 13.33 (18.00–71.00)	58.17 ± 6.46 (47.00–65.00)	57.67 ± 10.96 (18.00–71.00)	0.843
Ethnicity				0.232
Hispanic	2 (11.1%)	0 (0%)	2 (6.7%)	
Non-Hispanic	16 (88.9%)	12 (100%)	28 (93.3%)	
Race				0.408
African American	0 (0.0%)	1 (8.3%)	1 (3.3%)	
Asian: other	0 (0.0%)	1 (8.3%)	1 (3.3%)	
Black or African American: other	2 (11.1%)	0 (0.0%)	2 (6.7%)	
European Descent	7 (38.9%)	5 (41.7%)	12 (40.0%)	
Unknown	3 (16.7%)	1 (8.3%)	4 (13.3%)	
White: other	2 (11.1%)	3 (25.0%)	5 (16.7%)	
White: unknown	4 (22.2%)	1 (8.3%)	5 (16.7%)	
Gender, male	12 (66.7%)	8 (66.7%)	20 (66.7%)	1.000
HLA mismatch				0.597
0 of 0	13 (72.2%)	9 (75.0%)	22 (73.3%)	
0 of 2	0 (0.0%)	1 (8.3%)	1 (3.3%)	
0 of 6	1 (5.6%)	0 (0.0%)	1 (3.3%)	
1 of 6	1 (5.6%)	0 (0.0%)	1 (3.3%)	
3 of 10	1 (5.6%)	0 (0.0%)	1 (3.3%)	
No molecular	2 (11.1%)	2 (16.7%)	4 (13.3%)	
BMI, kg/m^2^ (range)	27.77 ± 5.03 (19.49–36.06)	30.12 ± 4.39 (22.93–36.44)	28.71 ± 4.85 (19.49–36.44)	0.198
Bridged with LVAD				0.654
No	9 (50.0%)	7 (58.3%)	16 (53.3%)	
Yes	9 (50.0%)	5 (41.7%)	14 (46.7%)	
Donor age, y (range)	29.00 ± 10.11 (18.00–52.00)	30.75 ± 13.74 (18.00–60.00)	29.70 ± 11.20 (18.00–60.00)	0.683
Blood type				0.265
A	6 (33.3%)	7 (58.3%)	13 (43.4%)	
AB	1 (5.6%)	2 (16.7%)	3 (10.0%)	
B	3 (16.7%)	1 (8.3%)	4 (13.3%)	
O	8 (44.4%)	2 (16.7%)	10 (33.3%)	
Hypertension				0.420
Unknown/miss	2 (11.1%)	2 (16.7%)	4 (13.3%)	
No	4 (22.2%)	4 (33.3%)	8 (26.7%)	
Yes	12 (66.67%)	6 (50.0%)	18 (60.0%)	
Diabetes				0.691
Unknown/miss	3 (16.7%)	1 (8.3%)	4 (13.4%)	
No	7 (38.9%)	6 (50.0%)	13 (43.4%)	
Yes	8 (44.4%)	5 (41.7%)	13 (43.3%)	
Time from transplantation, mo				0.640
Prerejection	2.52 ± 0.04	2.69 ± 0.28	2.62 ± 0.19	
Rejection	5.53 ± 0.40	6.16 ± 0.72	5.91 ± 0.47	
Postrejection	10.93 ± 0.77	9.60 ± 0.81	10.17 ± 0.59	
Immunosuppression				
Induction	17 (94.4%)	11 (91.7%)	28 (93.3%)	1.000
Basiliximab	5 (27.8%)	1 (8.3%)	6 (20.0%)	0.358
ATG	12 (66.7%)	10 (83.3%)	22 (73.3%)	0.419
Baseline immunosuppression at 3 mo				
Mycophenolate mofetil	17 (94.4%)	10 (83.3%)	27 (90.0%)	0.548
Azathioprine	1 (5.6%)	1 (8.3%)	2 (6.7%)	1.000
Tacrolimus	17 (94.4%)	10 (83.3%)	27 (90.0%)	0.548
Sirolimus	1 (5.6%)	0 (0.0%)	1 (3.3%)	1.000
Cyclosporin	0 (0.0%)	2 (16.7%)	2 (6.7%)	0.152
Prednisone	18 (100%)	12 (100%)	30 (100%)	
Baseline immunosuppression at 12 mo				
Mycophenolate mofetil	17 (94.4%)	9 (75.0%)	26 (86.7%)	0.274
Azathioprine	1 (5.6%)	2 (16.7%)	3 (10.0%)	0.548
Tacrolimus	12 (66.7%)	5 (41.7%)	17 (56.7%)	0.176
Sirolimus	8 (44.4%)	4 (33.3%)	12 (40.0%)	0.709
Cyclosporin	0 (0.0%)	2 (16.7%)	2 (6.7%)	0.152
Prednisone	10 (55.6%)	6 (50.0%)	16 (53.3%)	0.765

ATG, anti-thymocyte globulin; BMI, body mass index; LVAD, left ventricular assist device.

### DEGs During Rejection as Compared With Prerejection

#### Number of DEGs

**FIGURE 2.**
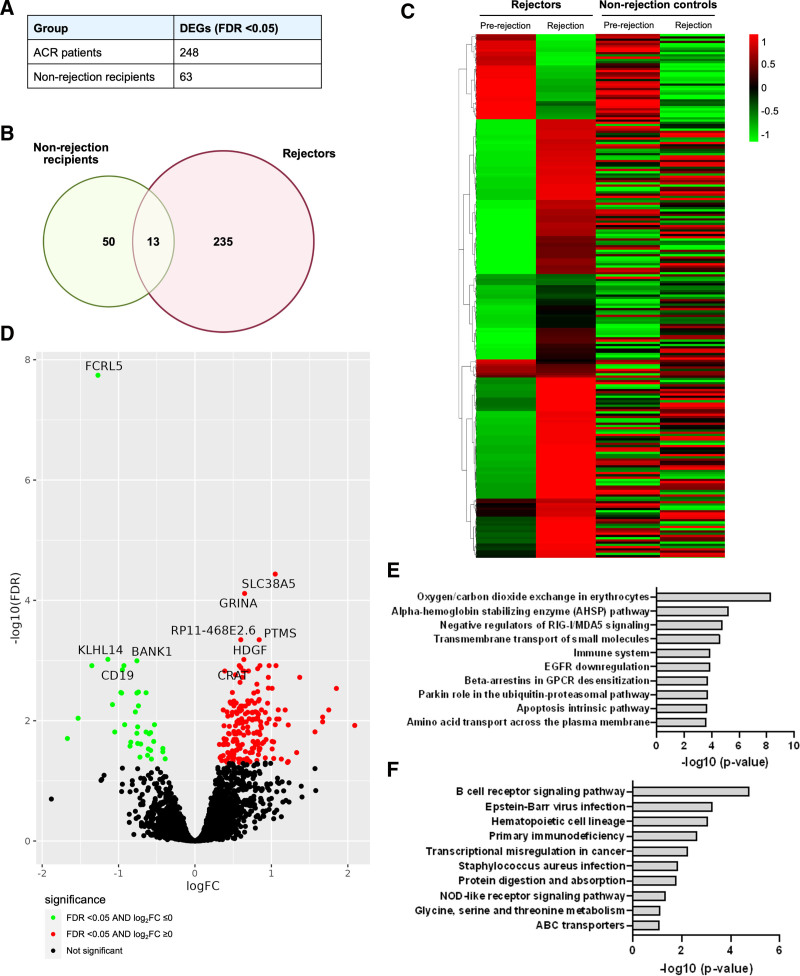
DEGs between timepoint of rejection and pre-rejection from ACR. A, DEGs with FDR <5%, 248 genes from ACR, 63 genes from NR. B, Overlaps of DEGs between ACR and NR. C, Heatmap for the 235 rejection-specific genes between ACR and NR. D, Volcano plots for the 235 rejection-specific genes, upregulated genes in red and downregulated genes in green, top 10 DEGs are shown. E, Significant pathways for 197 upregulated DEGs. F, Significant pathways for 38 downregulated DEGs. ACR, acute cellular rejection; DEG, differentially expressed gene; FDR, false discovery rate; NR, nonrejection.

To understand the process of underlying change in gene expression with rejection, we investigated DEGs between the timepoint of rejection compared with prerejection in the rejector group (comparison 1, Figure 1), resulting in the identification of 248 DEGs associated with ACR. There were only 63 DEGs in the control group during a similar period (Figure 2A). After excluding the 13 common genes that overlapped between the two groups, a set of 235 DEGs, described as rejection-specific genes which significantly change during the episode of rejection as compared with prerejection, are shown in a heatmap (Figure 2B and C).

#### Significant Upregulated and Downregulated DEGs

Among these rejection-specific genes, 197 DEGs were upregulated and 38 were downregulated. The volcano plot (Figure 2D) annotates representative top-ranking genes. The most significantly upregulated DEGs included *SLC38A5, GRINA, RP11-468E2.6, PTMS, HDGF*, and *CRAT* and are associated with the immune system, T-cell activation and differentiation, and apoptosis.^11-14^ Among the significantly downregulated DEGs, expression of several B cell-related genes (*FCRL5, KLHL14, BANK1,* and *CD19*) decreased, consistent with suppression of B-cell receptor signaling (Table 2).^15-18^

**TABLE 2.** List of 20 genes most significantly differentially expressed between timepoint of rejection and pre-rejection in ACR

	Gene symbol	Gene name	Function	log2FC
1	AQP1	Aquaporin 1	Water channel protein	1.06
2	SLC38A5	Solute Carrier Family 38 Member 5	Amino acid transmembrane transporter	1.05
3	TMC5	Transmembrane Channel Like 5	Ion transmembrane transport	0.97
4	NPRL3	NPR3 Like, GATOR1 Complex Subunit	GTPase activator activity	0.85
5	PTMS	Parathymosin	Mediate immune function	0.84
6	TMEM63B	Transmembrane Protein 63B	Osmosensitive calcium-permeable cation channel	0.82
7	YBX1	Y-Box Binding Protein 1	DNA and RNA binding protein	0.7
8	GRINA	Glutamate Ionotropic Receptor NMDA Type Subunit Associated Protein 1	Transmembrane transporter binding	0.65
9	HDGF	Heparin Binding Growth Factor	Mitogenic and DNA-binding activity	0.64
10	CDKL1	Cyclin Dependent Kinase Like 1	Protein tyrosine kinase activity	0.63
11	RP11-468E2.6	Long non-coding RNA		0.6
12	MAF1	MAF1 Homolog, Negative Regulator Of RNA Polymerase III	Negative effector of RNA polymerase III	0.6
13	CRAT	Carnitine O-Acetyltransferase	Enzyme for metabolic pathways	0.58
14	DCAF11	DDB1 And CUL4 Associated Factor 11	Substrate receptor	0.39
15	BANK1	B Cell Scaffold Protein With Ankyrin Repeats 1	B-cell activation	−0.76
16	CD19	CD19 Molecule	B-cell antigen receptor	−0.93
17	FCRL2	Fc Receptor Like 2	B-cell development and differentiation	−0.95
18	KLHL14	Kelch Like Family Member 14	Promotes ubiquitylation of B-cell receptor	−1.14
19	FCRL5	Fc Receptor Like 5	B-cell development and differentiation	−1.27
20	OSBPL10	Oxysterol Binding Protein Like 10	Glycerophospholipid biosynthesis and Metabolism	−1.35

ACR, acute cellular rejection.

#### Pathway Analysis

To uncover the biological processes and pathways associated with the genes of interest, we used the Enrichr tool (accessible at https://maayanlab.cloud/Enrichr) to perform gene ontology (GO) analysis and pathway enrichment analysis. GO analysis identified biological processes associated with nitrogen compound transport, carbon dioxide transport, and one-carbon compound transport (**Figure S1, SDC**, https://links.lww.com/TXD/A822). Pathway analysis of upregulated DEGs identified several important biological processes, including negative regulators of RIG-I, MDA5 signaling, immune system, adaptive immune system, signaling by NOTCH, negative regulators of DDX58/IFIH1 signaling, SMAD2/SMAD3:SMAD4 Heterotrimer regulates transcription pathway, regulation of nuclear factor kappa B (NF-κB) signaling, Nef-mediated CD8 downregulation, and signaling by transforming growth factor beta (TGF-β) receptor complex (Figure 2E). Interestingly, the downregulated DEGs exhibited significant enrichment in B-cell activation pathways and GO biologic processes (Figure 2F; **Figure S2, SDC,** https://links.lww.com/TXD/A822). This indicates potential alterations in B cell–related functions and adaptive immunity in the context of rejection.

#### PPI Analysis

The DEGs associated PPI was assessed using Cytoscape and stringApp with not >20 interactors and leading to development of an enrichment map. Similar results were found in the enrichment map with upregulated genes mapping to important domains of NF-κB signaling, toll-like receptor 3, 4, 7, and 8 cascade, MAP kinase, FCER1 mediated activation of NF-κB, MyD88 cascade, NOTCH1 signaling, IL-1 signaling, activation of NF-κB in B cells, TGF-β, and SMAD signaling (**Figure S3, SDC**, https://links.lww.com/TXD/A822). Downregulated genes in enrichment map signified interaction in domains of B-cell differentiation, B-cell receptor signaling, SH3 and SH2 domain, interleukin (IL)-5, IL-3 signaling, GPVI activation cascade, FCERI-mediated MAPK activation (**Figure S4, SDC**, https://links.lww.com/TXD/A822).

### DEGs Between the Timepoint of Rejection and Postrejection

#### Number of DEGs

**FIGURE 3.**
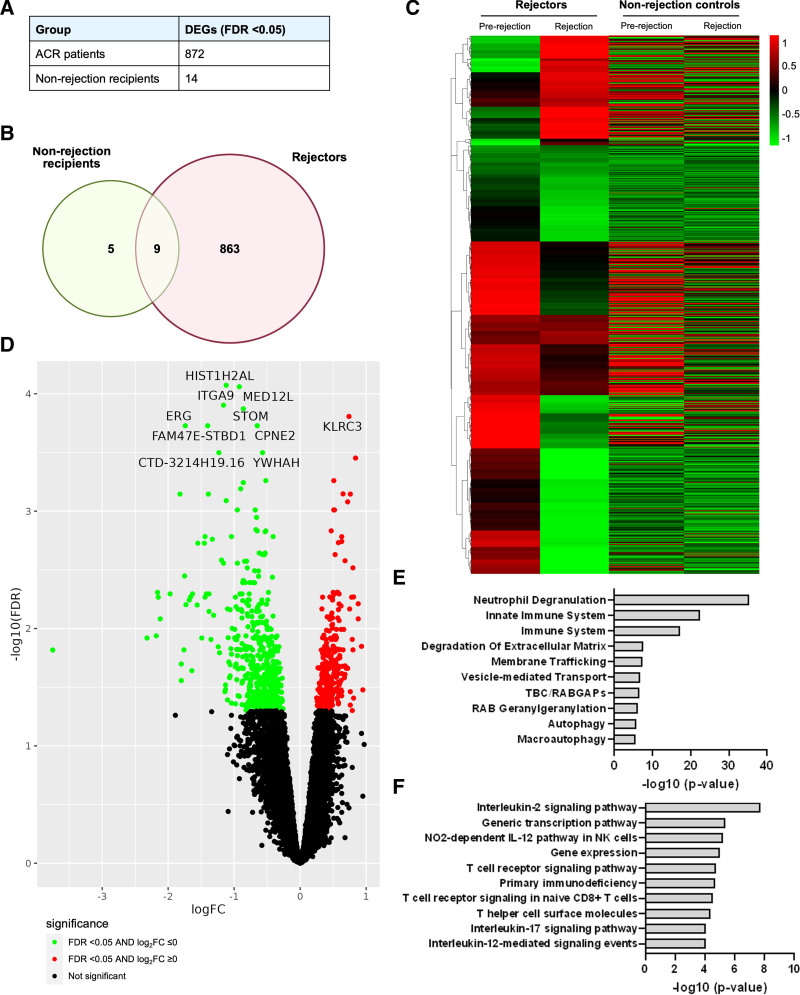
DEGs between timepoint of rejection and postrejection from ACR. A, DEGs with FDR <5%, 872 genes from ACR, and 14 genes from NR. B, Overlaps of DEGs between ACR and NR. C, Heatmap for the 863 postrejection genes between ACR and NR. D, Volcano plots for the 863 postrejection genes, upregulated genes in red and downregulated genes in green, top 10 DEGs are shown. E, Significant pathways for 327 upregulated DEGs. F, Significant pathways for 536 downregulated DEGs. ACR, acute cellular rejection; DEG, differentially expressed gene; FDR, false discovery rate; NR, nonrejection.

To understand the biological processes that get activated after rejection, we also performed a comparison of DEG between the timepoint of postrejection and rejection (comparison 2, Figure 1). There were 872 genes that were differentially expressed in the rejector group, and only 14 genes in the controls (Figure 3A). After excluding 9 overlapping genes between the 2 groups, we identified a set of 863 unique DEGs, which significantly change after rejection as shown in the heatmap (Figure 3B and C).

#### Significant Upregulated and Downregulated DEGs

Among these postrejection DEGs, 327 were upregulated and 536 were downregulated. The volcano plot (Figure 3D) annotates the top 10 DEGs between postrejection and rejection in the ACR group. The most significantly upregulated DEGs were *KLRC3* and *KLRC4,* which are NK-cell receptors that recognize HLA-E/major histocompatibility complex (MHC) class I.^19,20^ The most significantly downregulated DEGs included nucleosome/chromatin (*HIST1H2AL*), transcriptional regulation (*MED12L, ERG*), cell adhesion/ion transport (*ITGA9*, *STOM*, *CPNE2*), and signaling (*YWHAH*) (Table 3).^21-28^

**TABLE 3.** List of 20 genes most significantly differentially expressed between timepoint of rejection and postrejection in ACR

	Gene symbol	Gene name	Function	log2FC
1	KLRC4	Killer Cell Lectin Like Receptor C4	Receptor for the recognition of MHC class I	0.84
2	GPR171	G Protein-Coupled Receptor 171	Negative regulator T-cell function	0.76
3	KLRC3	Killer Cell Lectin Like Receptor C3	Receptor for the recognition of MHC class I	0.74
4	ZNF625	Zinc Finger Protein 625	Transcriptional regulation	0.72
5	THEMIS	Thymocyte Selection Associated	Positive and negative T-cell selection	0.65
6	C19orf12	Chromosome 19 Open Reading Frame 12	Small transmembrane protein	0.51
7	ZDHHC3	Zinc Finger DHHC-Type Palmitoyltransferase 3	Calcium transporter	−0.52
8	YWHAH	Tyrosine 3-Monooxygenase/Tryptophan 5-Monooxygenase Activation Protein Eta	Mediate signal transduction	−0.57
9	CPNE2	Copine 2	Calcium-mediated intracellular processes	−0.65
10	STOM	Stomatin	Regulates ion channel activity and transmembrane ion transport	−0.86
11	MYBL2	MYB Proto-Oncogene Like 2	Regulation of cell survival, proliferation, and differentiation	−0.86
12	MSRB3	Methionine Sulfoxide Reductase B3	Enzyme acting as a monomer and requires zinc as a cofactor	−0.9
13	MED12L	Mediator Complex Subunit 12L	Transcriptional coactivation of nearly all RNA polymerase II-dependent genes	−0.92
14	HIST1H2AL	H2A Clustered Histone 16	Transcription regulation, DNA repair, DNA replication and chromosomal stability	−1.12
15	NOS1AP	Nitric Oxide Synthase 1 Adaptor Protein	A cytosolic protein	−1.12
16	ITGA9	Integrin Subunit Alpha 9	A receptor for VCAM1, cytotactin and osteopontin	−1.16
17	CTD-3214H19.16	CTD-3214H19.16	Predicted intracellular protein	−1.23
18	FAM47E-STBD1	FAM47E-STBD1 Readthrough		−1.39
19	ERG	ETS Transcription Factor ERG	Transcriptional regulator	−1.74
20	HP	Haptoglobin	Elimination of free hemoglobin and the neutralization of oxidative damage	−1.82

ACR, acute cellular rejection; MHC, major histocompatibility complex; VCAM1, vascular cell adhesion molecule-1.

#### Pathway Analysis

Pathway enrichment analysis for the 536 downregulated genes identified pathways associated with neutrophil degranulation, innate immune system, immune system, toll receptor cascades, MHC class II antigen presentation, PIK3C1/AKT pathway, calcineurin-dependent NFAT signaling, and TGF-β regulation of extracellular matrix (Figure 3E). GO biologic process showed significant enrichment in positive regulation of myeloid cell differentiation (**Figure S5, SDC**, https://links.lww.com/TXD/A822). These enrichment analyses signify the importance of decreased expression of inflammation and innate immunity in the postrejection phase. Pathway enrichment analysis for the 327 upregulated DEGs shows extensive enrichment in pathways of immunoregulatory interactions between lymphoid and nonlymphoid cells, and a pathway in adaptive immunity with specific role in response of cells of lymphoid tissues. IL-2 signaling pathway, NO_2_-dependent IL-12 pathway in NK cells, multiple T-cell receptor signaling pathways, and IL-17 signaling were also involved (Figure 3F). Similar enrichment was seen in GO in multiple T-cell and NK-cell pathways, cellular response to lectin and lectin receptor signaling (**Figure S6, SDC,** https://links.lww.com/TXD/A822).

#### PPI Analysis

In Cytoscape/EnrichmentMap, postrejection downregulated genes clustered in TLR3/MyD88 cascades, TGF-β/SMAD signaling, innate immune pathways (neutrophil degranulation, myeloid leukocyte activation), and platelet and azurophilic granule lumen, many of which were upregulated at the time of rejection (**Figure S7, SDC,** https://links.lww.com/TXD/A822). Conversely, postrejection upregulated genes mapped to leukocyte differentiation/adhesion and to T- and B-cell receptor signaling with signatures consistent with T-cell lymphopenia (reduced CD3^+^/CD8^+^) but increased NK cell cytotoxicity (**Figure S8, SDC,** https://links.lww.com/TXD/A822).

### Dynamic Map of Common Transcriptomic Changes in ACR

**FIGURE 4.**
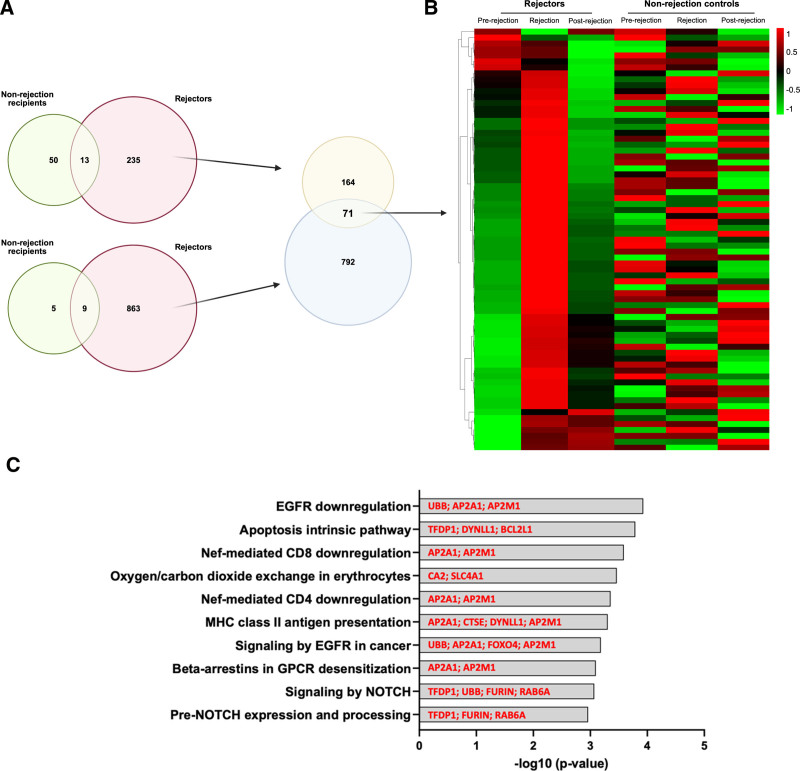
Dynamic of transcriptomic for ACR. A, Overlaps of DEGs between 235 rejection-specific genes and 863 postrejection genes. B, Heatmap for the 71 genes illustrating the dynamic of transcriptomic for ACR. C, Significant pathways for 71 genes. ACR, acute cellular rejection; DEG, differentially expressed gene.

To identify genes that span the dynamic spectrum of the entire ACR process, DEGs that overlapped the 235 rejection-specific and 863 postrejection genes were identified. There were 71 genes that significantly changed both at the timepoint of rejection and postrejection (Figure 4A). Interestingly, most of these 71 genes get upregulated during rejection and get downregulated postrejection (Figure 4B). Pathway enrichment analysis for these genes identified CD4 and CD8, MHC class II antigen presentation and NOTCH signaling pathways (Figure 4C).

### Prediction of ACR Using DEGs

**FIGURE 5.**
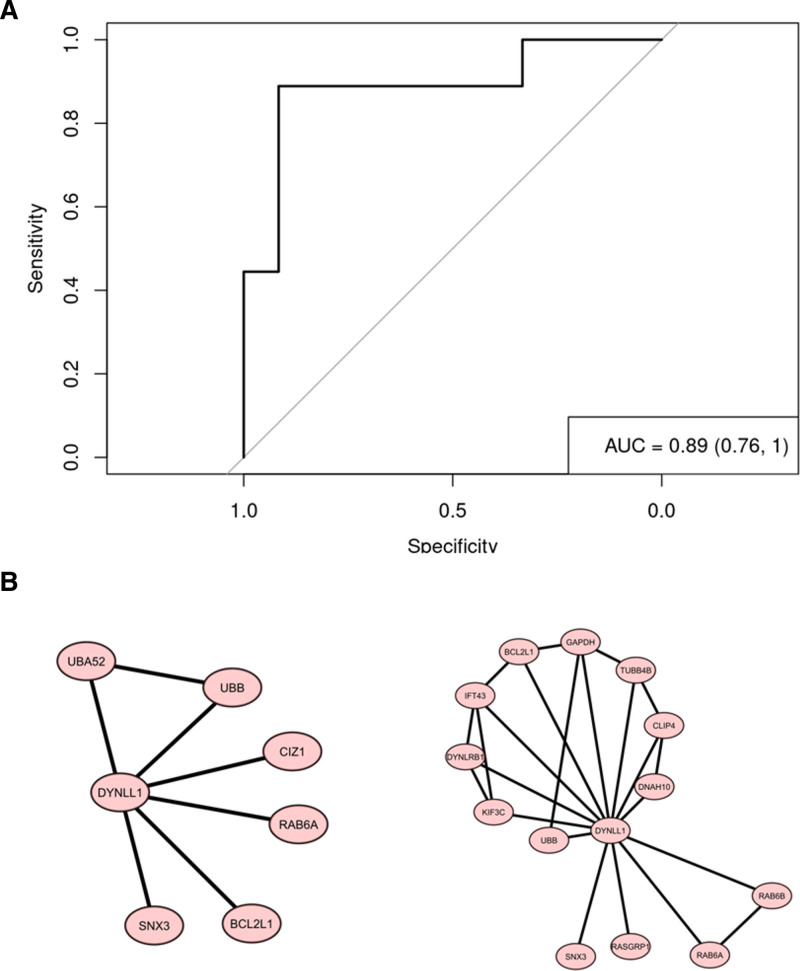
Rejection predicting gene. PPI map showing the DEGs interacted with *DYNLL1*. DEG, differentially expressed gene; PPI, protein–protein interaction.

The 235 DEGs at the time of rejection were used to predict rejection. Using elastic net and 5-fold cross-validation for identifying this variable, “*DYNLL1*” and “*SERF2*” remained in the model. Subsequent variable selection resulted in the identification of one rejection predictive gene *DYNLL1* (odds ratio 2.26; 95% confidence interval, 1.24-4.43; *P* = 0.028) and another gene *SERF2* (protein aggregation and toxicity) which were used in the logistic regression model resulting in a cross-validated receiver operating characteristic of 0.63 (Figure 5A). The PPI map of the DEGs with *DYNLL1* was assessed at both the rejection and postrejection timepoints resulting in the identification of interacting proteins encoded by *SNX3*, *UBB*, *BCL2L1*, and *RAB6A* which are known to be involved in intracellular trafficking, association with MyD88 cascade, antagonizing apoptosis, and association with the innate immune system respectively (Figure 5B).

## DISCUSSION

Although the morbidity of rejection after HTx has declined in recent years because of effective immunosuppressive therapy, it remains the primary cause of recipient retransplantation and death. ACR has been reported to be associated with transcriptional changes in resident cells or changes in immune cells, such as effector T cells, macrophages, and NK cells within heart tissue.^29-34^ Targeted (20 genes) transcriptomic profiling of peripheral blood mononuclear cells in HTx recipients with ACR that initially used microarray technology and subsequently reverse-transcription PCR reflects these tissue changes and has provided an alternative to EMB to diagnose rejection. However, there are limited data on peripheral blood whole genome transcriptomic profiling in adult HTx recipients that could reveal insights into the pathogenesis of ACR and could potentially lead to identification of new noninvasive biomarkers of ACR in an agnostic manner.

In this study, we performed whole genome transcriptomics using longitudinal peripheral blood sample analysis which allows for each patient with ACR to serve as their own controls thus providing greater power and in addition the longitudinal sample analysis in controls allowed for adjustment of confounding changes that can be temporally related to transplant. A pattern of upregulation involving the innate and adaptive immune system pathways and downregulation of B-cell pathway related genes was identified during ACR. Specific antigens presented by the transplanted tissue lead to the activation and proliferation of T cells, which in turn could lead to a decrease in the expression of genes related to B-cell development and function. It is important to note that immune responses are complex and can involve various interactions and regulatory mechanisms.^35-38^ In addition, a larger number of genes (n = 863) significantly changed in expression postrejection as compared with during ACR (n = 235), as opposed to expectedly only 14 genes temporally changing expression in the control group. The change in gene expression postrejection may not only reflect immune adaptive and reparative processes but also treatment related effects that involved IL-2 and TGF-β signaling, and the innate immune system that ultimately could impact long-term outcomes. For example, IL-2 plays an important role in the generation of memory T cells and TGF-β can result in reparative cardiac fibrosis.^39,40^

We also used a logistic regression classifier for predicting patients with rejection status and found *DYNLL1* as a significant predictor gene of ACR. The protein encoded by *DYNLL1* (cytoplasmic dynein light chain 1) acts as a motor for the intracellular retrograde motility of vesicles and organelles along microtubules. It may play a role in changing or maintaining the spatial distribution of cytoskeletal structures.^41^ It also has been reported to play an important role in TLR4-mediated T cell–independent antibody response and has been found to be essential for TLR4- and IL-1-mediated activation of NF-κB and MAPK in B cells. TLR and IL-1 converge on MyD88 for activation of NF-κB and MAPK.^42^ Our study potentially highlights the important role of TLR-mediated innate immune response requiring *DYNLL1*, in addition to T cell followed by B-cell involvement. This hypothesis is concordant with studies describing the role of not just cellular but innate immunity as well.^41^ In a study that identifying circulating extracellular vesicles (EVs) as noninvasive biomarkers of rejection in heart transplant, *DYNLL1* was identified as one of main interactors with EV surface markers in ACR indicating its potential role in cardiac allograft rejection.^43^ The AUC of 0.63 for the model using *DYNLL1* and *SERF2* expression is comparable to the reported performance of the AlloMap genomic expression profiling test (AUC 0.69).^44^ The AUC for AlloSure cell-free DNA test to detect cardiac rejection varies from 0.61 to 0.69 based on the patient population studied and clinical indication for testing.^45^

Our study has several limitations. A relatively small sample size was studied (18 patients with rejection) that reflects the infrequent occurrence of rejection during routine surveillance, logistical challenges of prospective patient recruitment and longitudinal blood sample collection, which, however, in turn enhanced the robustness and temporal resolution of our analysis. Transcriptomic analysis of paired endomyocardial biopsy tissue specimens was not performed which prevents us from confirming that peripheral signatures reflect intragraft processes. However, prior studies have shown that peripheral blood transcriptomic signatures correlate with rejection and clinical outcomes,^2^ supporting their use as reliable surrogates for intragraft immune activity. Our focus was on analyzing systemic, noninvasive, blood-based transcriptomic signals that would more accurately reflect the overall immune response during ACR rather than changes in the cardiac allograft. Our study was not intended to develop clinically actionable biomarkers for detecting rejection; rather, it was designed to provide a comprehensive characterization of the systemic biological changes underlying the rejection process. Although we identified gene signatures associated with rejection risk, these findings are exploratory in nature and would require validation in larger, prospective, multicenter studies to ensure reproducibility, generalizability, and clinical utility.

In conclusion, this whole genome blood transcriptomic study describes not only the temporal changes that occur broadly in the expression of the circulating peripheral white blood cell population but also provides insight into the early pathogenesis of ACR and its subsequent immune and reparative processes using an agnostic approach. The abundant, dynamic peripheral transcriptomic changes during ACR and a relative absence of these in comparable patients without ACR also highlight the potential role of *DYNLL1* in innate immunity. These hypothesis generating findings provide a foundation for investigating whether modulating identified pathways could influence rejection outcomes and whether integrating transcriptomic data might yield clinically useful tools.

## ACKNOWLEDGMENTS

The authors thank the Mayo Clinic Transplant Center, Department of Cardiovascular Medicine and Center for Individualized Medicine for supporting this study.

## Supplementary Material


