# Supplementary material for: Longitudinal Peripheral Blood Transcriptomics Reveal Novel Signatures During Cardiac Allograft Rejection
Source: Transplant Direct. 2026 Jan 20;12(2):e1882. doi: 10.1097/TXD.0000000000001882 (PMC12818864; doi:10.1097/TXD.0000000000001882)

Figure S1. GO biological process for 197 upregulated DEGs identified from comparison between timepoint of rejection to pre-rejection.

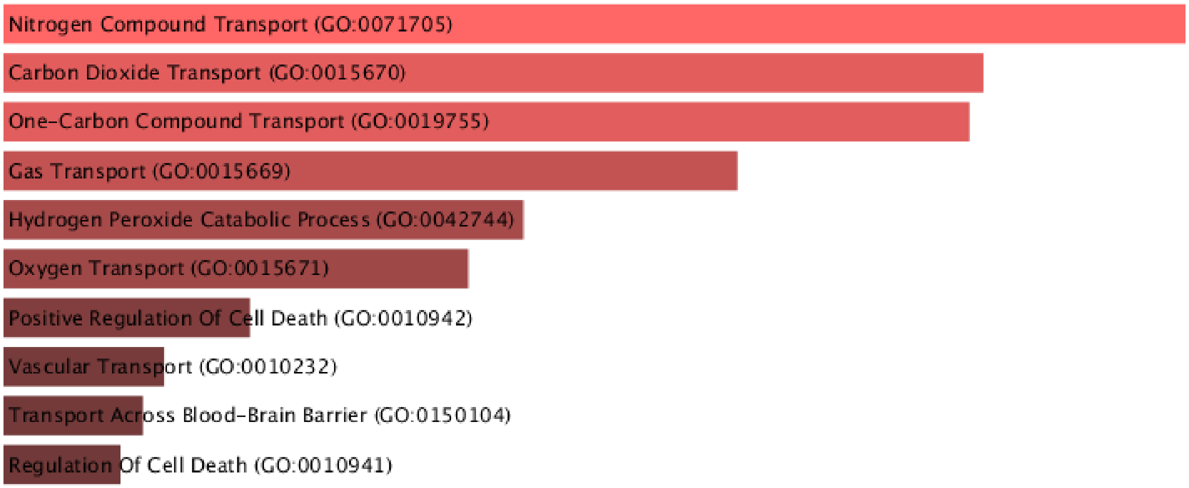

Figure S2. GO biological process for 38 downregulated DEGs identified from comparison between timepoint of rejection to pre-rejection.

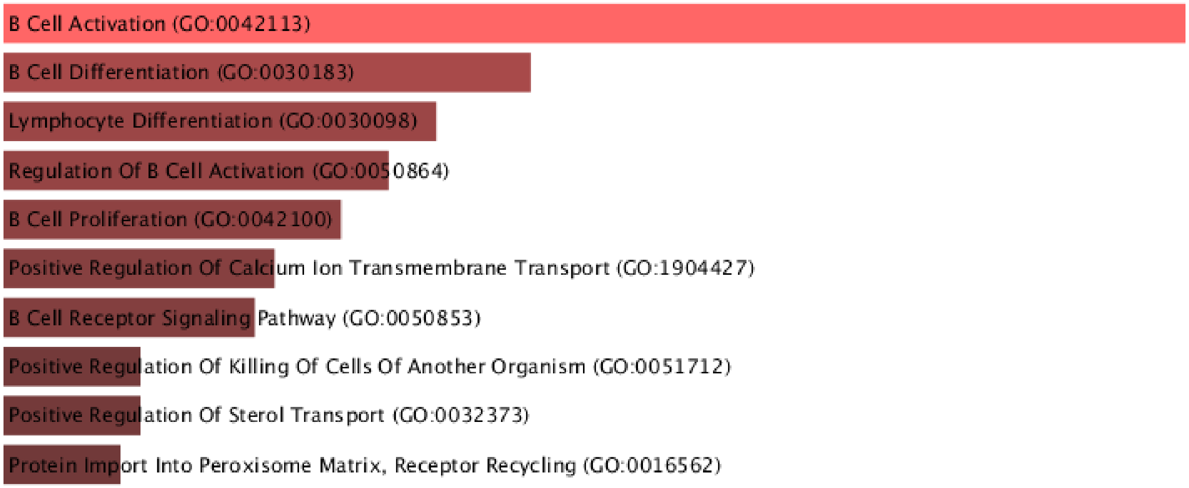

Figure S3. PPI enrichment derived from 197 upregulated DEGs identified from comparison between timepoint of rejection to pre-rejection. Nodes: group of genes enriched in one pathway. Edges: overlap of the genes between pathways.

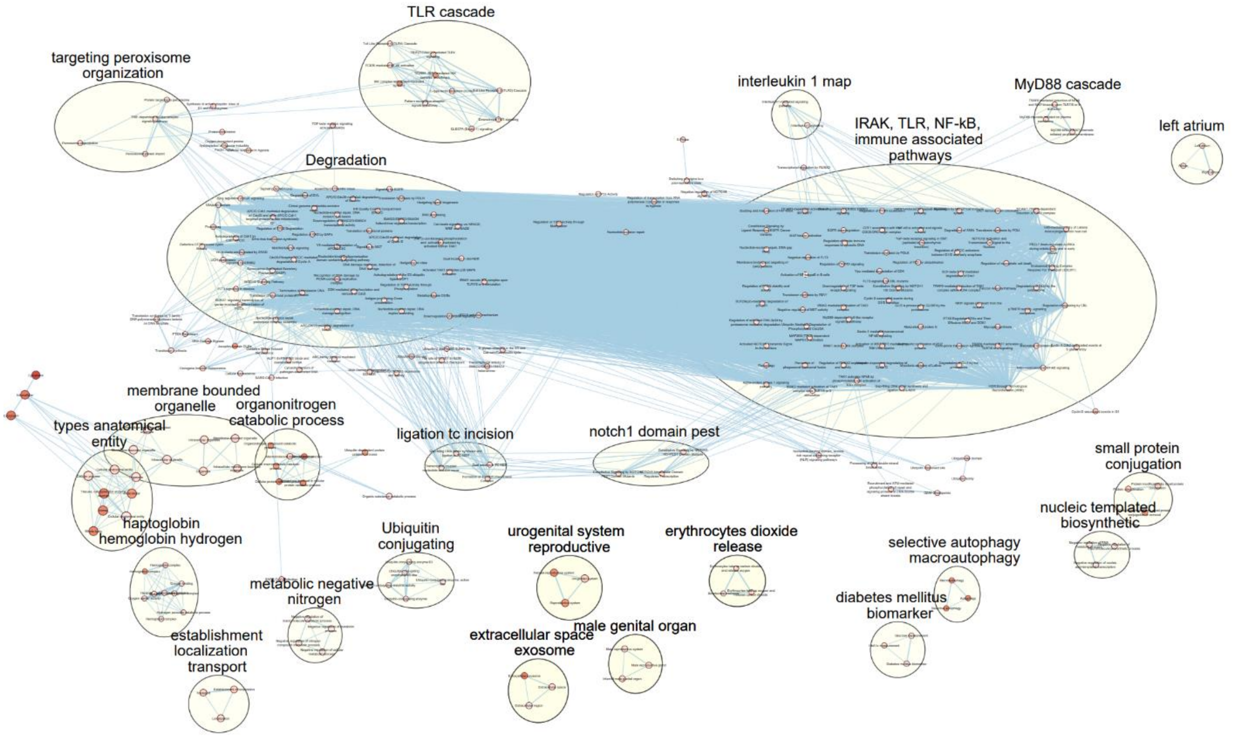

Figure S4. PPI enrichment derived from 38 downregulated DEGs identified from comparison between timepoint of rejection to pre-rejection. Nodes: group of genes enriched in one pathway. Edges: overlap of the genes between pathways

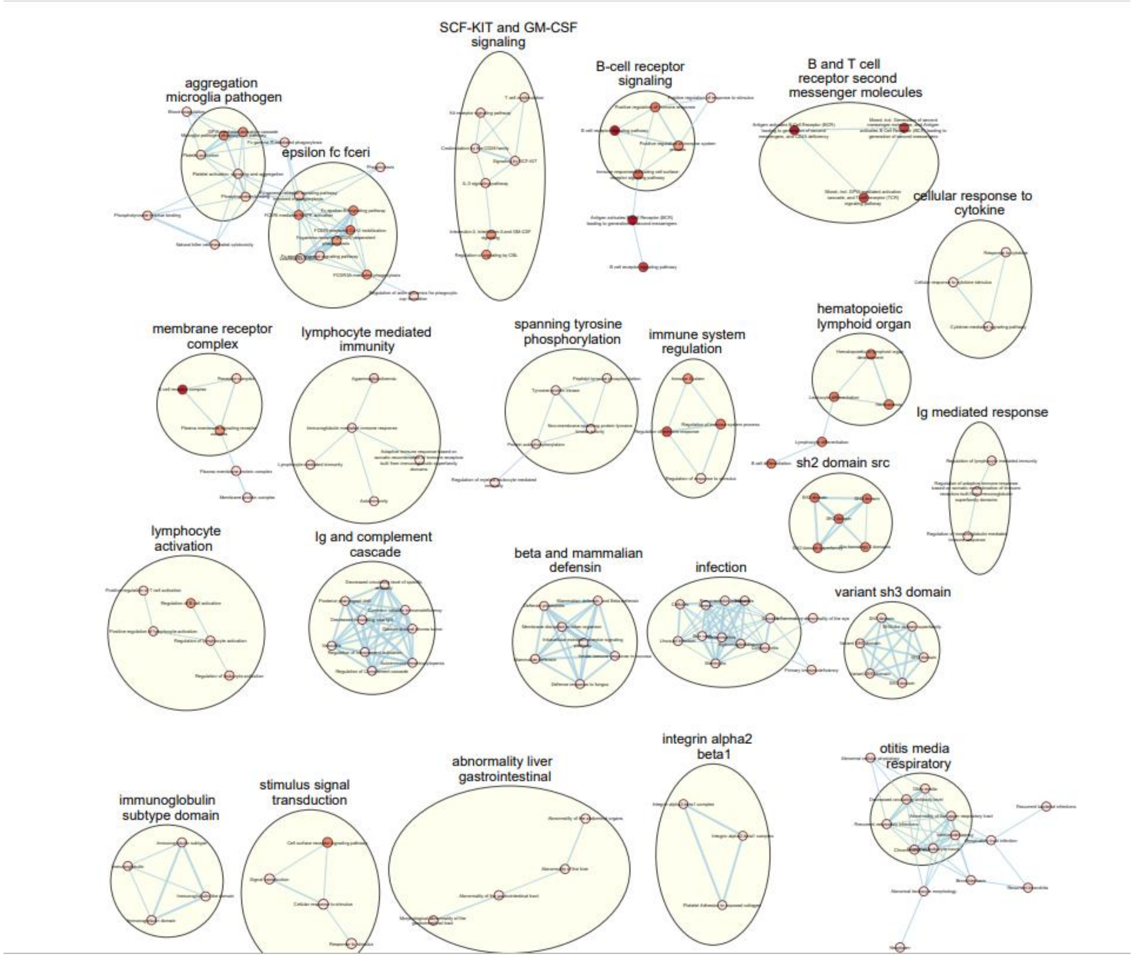

Figure S5. GO biological process for 536 downregulated DEGs identified from comparison between timepoint of post-rejection to rejection.

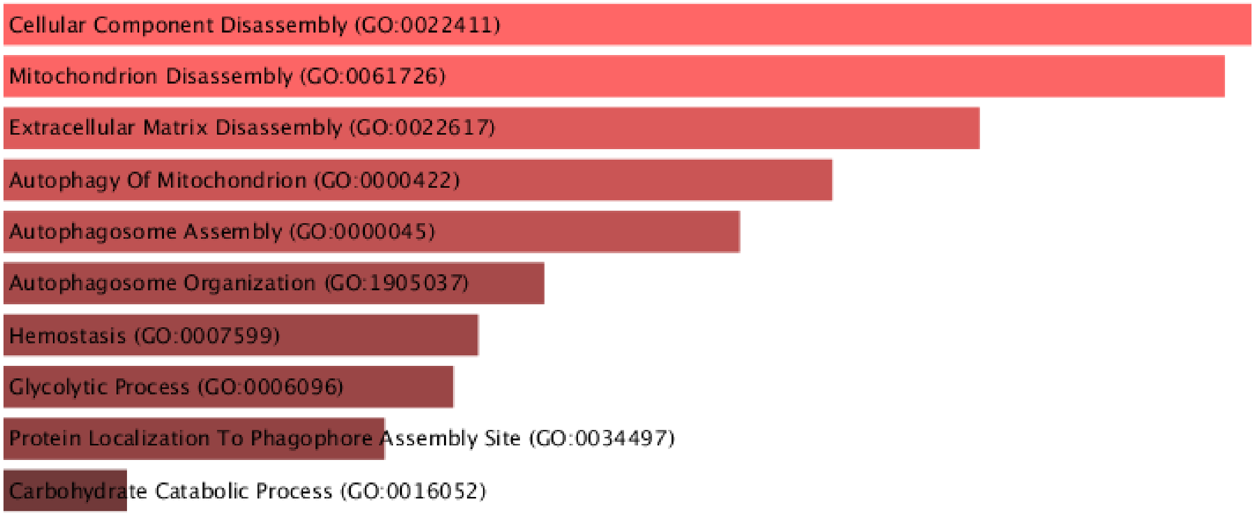

Figure S6. GO biological process for 327 upregulated DEGs identified from comparison between timepoint of post-rejection to rejection.

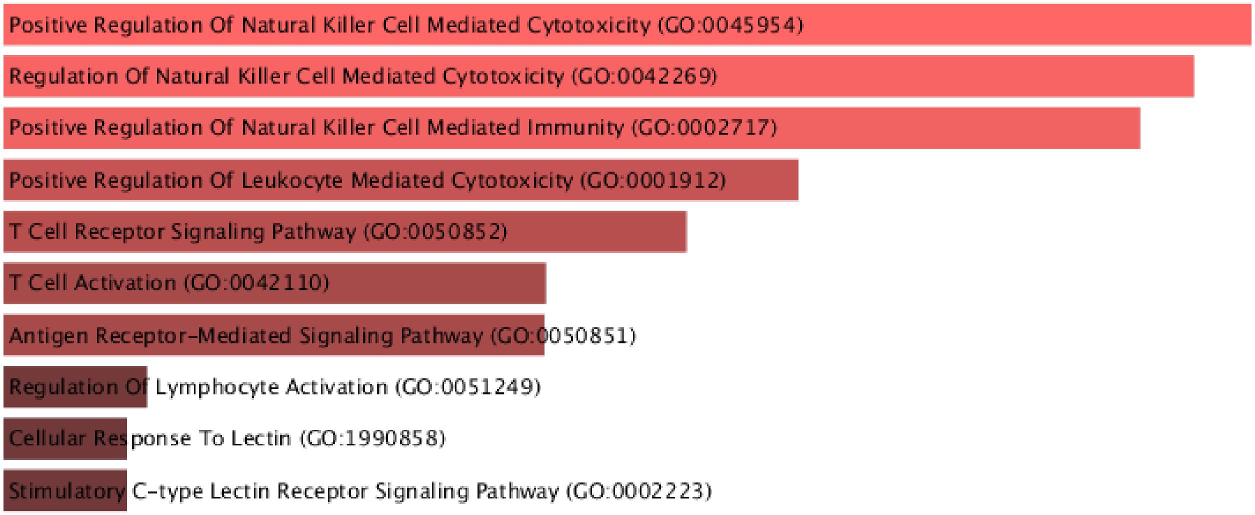

Figure S7. PPI enrichment derived from 327 downregulated DEGs identified from comparison between timepoint of post-rejection to rejection. Nodes: group of genes enriched in one pathway. Edges: overlap of the genes between pathways.

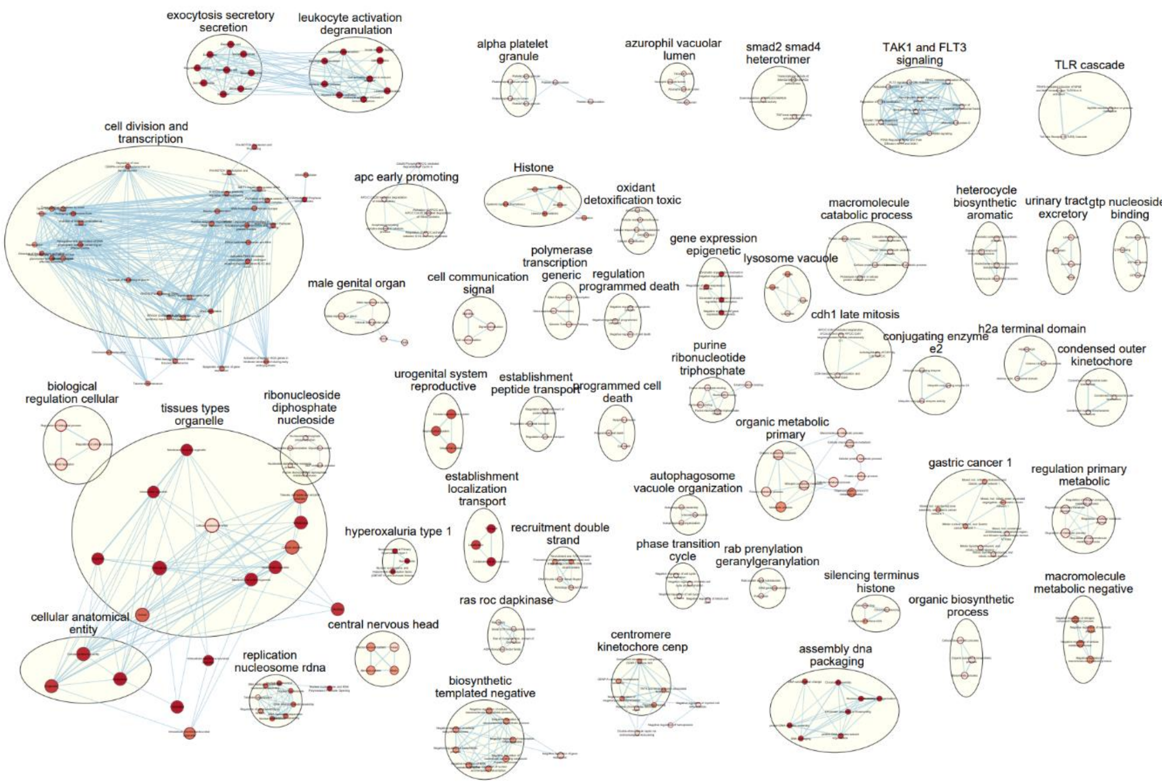

Figure S8. PPI enrichment derived from 536 upregulated DEGs identified from comparison between timepoint of post-rejection to rejection. Nodes: group of genes enriched in one pathway. Edges: overlap of the genes between pathways

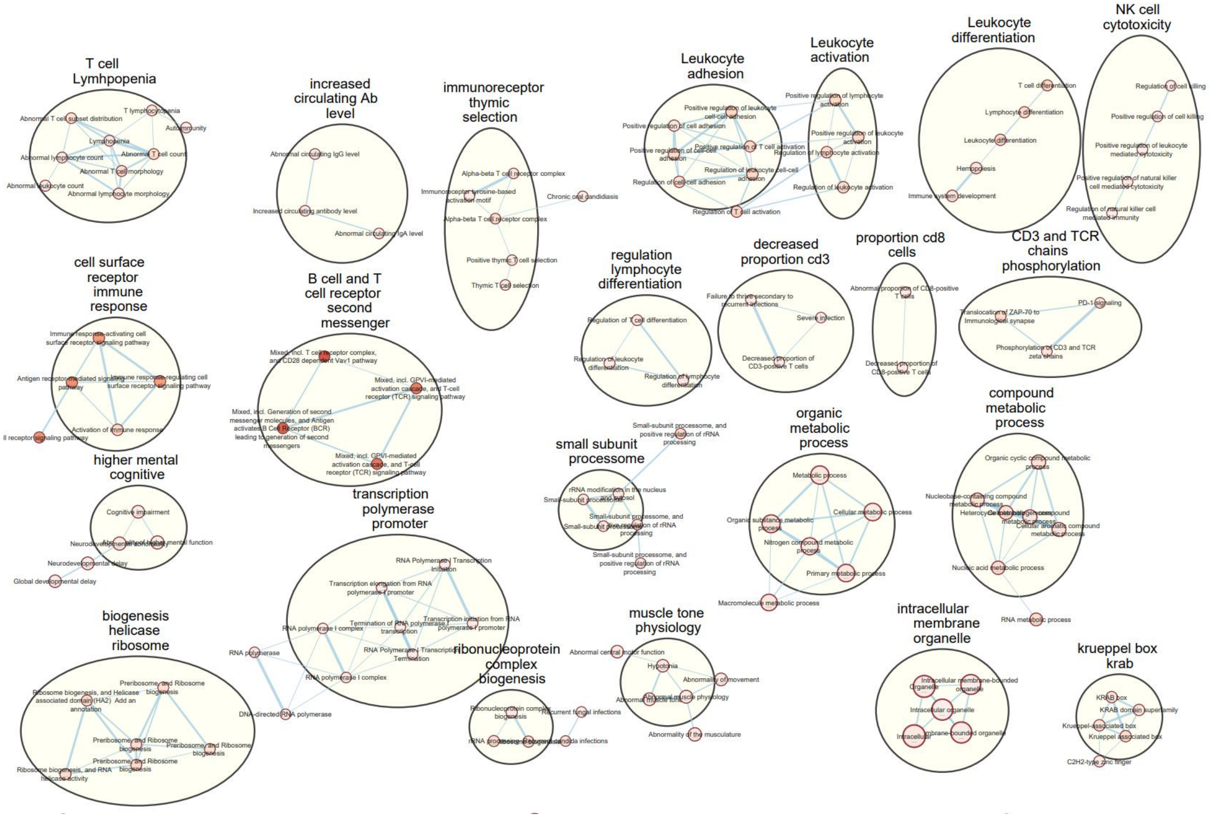

Supplement: Supplementary file 1 [file txd-12-e1882-s001.pdf]
